# Fine Motor Skills and Later Vocabulary Development Before and During the COVID-19 Pandemic: Evidence from Japanese Childcare Settings

**DOI:** 10.3390/children13070920

**Published:** 2026-07-12

**Authors:** Maiko Shigeeda, Smarika Shrestha, Munenori Matsumoto, Akihiro Kakuda, Hilda Meriyandah, Yanlin Wang, Afsari Banu Alpona, Yuri Nurdiantami, Yuko Sawada, Tokie Anme

**Affiliations:** 1 Graduate School of Comprehensive Human Sciences, University of Tsukuba, Tsukuba 305-8575, Japan; s2330403@u.tsukuba.ac.jp (M.S.);; 2 Faculty of Nursing, Reiwa Health Sciences University, Fukuoka 811-0213, Japan; 3 Department of Physical Therapy, Morinomiya University of Medical Sciences, Osaka 559-8611, Japan; 4 Faculty of Medicine, University of Tsukuba, Tsukuba 305-8575, Japan; 5 Faculty of Health Sciences, Universitas Pembangunan Nasional Veteran Jakarta, Jakarta 12450, Indonesia

**Keywords:** early childhood development, ecological theory, motor development, language development, COVID-19

## Abstract

**Highlights:**

**What are the main findings?**

- Fine motor skills predicted later vocabulary development both before and during the COVID-19 pandemic.
- The motor–language association differed between pre-pandemic and pandemic cohorts.

**What are the implications of the main findings?**

- Fundamental developmental pathways linking motor and language skills remained evident despite pandemic-related restrictions.
- Social and communicative opportunities may influence how motor experiences support language development.

**Abstract:**

Background/Objective: Early fine motor skills are consistently associated with later language development, but it remains unclear as to whether this relationship is maintained among children showing early development during the COVID-19 pandemic. In this study, we investigated whether the longitudinal association between early fine motor skills and subsequent vocabulary development differed between children demonstrating early development before and during the COVID-19 pandemic. Method: The participants were 1150 children (pre-pandemic cohort: *n* = 703; pandemic cohort: *n* = 447) attending licensed childcare centers in Japan. Hierarchical multiple regression and moderation analyses were conducted to examine whether fine motor skills at Time 1 (T1) predicted vocabulary at Time 2 (T2) after adjusting for baseline vocabulary, demographic characteristics, and home-rearing environment. Results: Fine motor skills at T1 significantly predicted vocabulary at T2 in both cohorts. A significant interaction between cohort and fine motor skills was observed (*B* = −0.09, *p* = 0.041), indicating that the strength of this association differed between the pre-pandemic and pandemic cohorts. Children in the latter demonstrated lower vocabulary outcomes across most levels of fine motor proficiency, with cohort differences becoming more pronounced with higher levels of baseline fine motor skills. Conclusions: The findings suggest that the fundamental association between fine motor skills and vocabulary development remained evident despite pandemic-related restrictions; however, changes in children’s social and communicative environments may have reduced opportunities to translate motor competencies into linguistic gains. These results highlight the importance of supportive social interactions and communicative experiences in facilitating early developmental processes.

## 1. Introduction

The COVID-19 pandemic profoundly altered children’s daily lives by reducing opportunities for physical activity and social interaction, alongside the introduction of preventive measures such as masking and social distancing. Early childhood is a particularly sensitive period in which motor and language skills develop rapidly [1,2,3,4]. From the perspective of ecological systems theory, development does not occur in isolation but is shaped by continuous interactions within a child’s immediate environment, including with family members, childcare professionals, and peers. In this regard, as core microsystems, home and childcare settings play a central and formative role in shaping a child’s developmental trajectory [5,6,7,8].

Unlike many other countries where childcare centers serving preschool-aged children were closed for prolonged periods due to COVID-19, Japan adopted a distinct policy response. While the government closed primary and secondary schools during the 2020 State of Emergency, childcare centers were, in principle, encouraged to remain operational to sustain children’s essential societal functions. However, this does not imply that their activities continued uninterrupted or were unchanged after this date; most local municipalities strongly recommended that parents keep their children at home whenever feasible. This created a paradoxical context in Japan: although childcare centers were technically accessible, many children, in practice, spent substantially more time within the domestic microsystem than they did before the pandemic [9].

To sustain this continuity, the classroom environment was transformed into a highly regulated and precautionary space. The school day was restructured around stringent protective routines, including frequent hand hygiene practices, continuous disinfection of shared materials by childcare professionals, “mokushoku” (silence during eating: a practice of requiring children to eat meals quietly without conversation with peers to minimize viral transmission) implementation, and acrylic partition installation. Additionally, events at childcare centers, such as sports days and performances, were modified, with singing and cheering restricted to comply with infection control measures [9,10].

Early childhood is a critical period during which fine motor and language abilities develop rapidly and interact to support children’s overall development [1,2,3,4]. Fine motor skills enable children to manipulate objects, explore their surroundings, and participate in play and daily activities, thereby promoting cognitive and learning experiences [3]. Expressive vocabulary, which reflects children’s ability to produce and use words meaningfully, is essential for communication, social interaction, and later academic success [7,8,11]. Increasing evidence suggests that motor and language development are linked through a close motor–language association rather than representing independent developmental processes. As children’s fine motor skills improve, they engage more actively in object exploration, joint attention, and social interactions, creating opportunities for caregivers and educators to provide responsive verbal input that supports vocabulary acquisition [12,13,14,15,16,17,18]. However, both fine motor and language development are influenced by multiple ecological factors, including the home-rearing environment, caregiver–child interactions, childcare experiences, and broader social contexts [5,6,7,8,19,20,21]. Therefore, understanding these developmental relationships is important for identifying how environmental changes, such as those experienced during the COVID-19 pandemic, may influence children’s developmental trajectories.

As fine motor abilities become more refined, children engage more actively with their surrounding environment through object manipulation, drawing, and tool use, creating opportunities for learning and social interaction [11,12]. From a developmental cascade perspective, motor and language development are considered reciprocal processes that influence each other over time, with one proposed mechanism underlying this association being that increasing fine motor competence expands opportunities for exploration and interaction with caregivers and the environment, thereby facilitating vocabulary development [12,13,14,15,16,17,18].

Although previous studies have documented the motor–language association [22,23,24,25,26,27], relatively little is known about whether it remains stable under major disruptions to children’s everyday experiences. Pandemic-related studies primarily focused on changes within individual developmental domains [28,29,30], whereas little is known about whether the motor–language association was also affected by the pandemic, which substantially impacted opportunities for social interaction, exploration, and participation in community settings [31,32]. Such changes may influence the processes through which motor experiences support language development; therefore, examining the longitudinal association between fine motor skills and vocabulary before and during the pandemic may provide insight into how the motor–language association operates under different ecological conditions.

In this study, we examined whether the longitudinal association between fine motor skills and vocabulary differed between children in pre-pandemic and pandemic cohorts. By comparing those who experienced early development under different social and environmental conditions, we investigated whether the strength of the association between early fine motor skills and later vocabulary development varied across different developmental contexts. Given the substantial disruptions to opportunities for exploration and social interaction during the pandemic, we hypothesized that this association would be weaker in the pandemic than in the pre-pandemic cohort.

## 2. Method

### 2.1. Study Design and Data Source

For this comparative longitudinal study, we used data from the Childcare Cohort Study (CCC), an ongoing nationwide longitudinal study conducted in licensed childcare facilities across multiple regions of Japan. Participating centers voluntarily enrolled in the developmental assessment program and provided anonymized data for the CCC, which collects annual information on children’s development and home-rearing environment.

In the present study, we compared two CCC cohorts: a pre-pandemic (2017–2019; *n* = 703; age range = 12–55 months; *M* = 32.71 months, *SD* = 12.48) and a pandemic cohort (2021–2023; *n* = 447; age range = 12–56 months; *M* = 35.44 months, *SD* = 11.66), yielding a total analytical sample of 1150 children. Data were obtained from 11 licensed childcare centers in the pre-pandemic cohort and 9 in the pandemic cohort.

Children assessed in 2020 were excluded because this year represented a transitional period during the onset of the COVID-19 pandemic, during which public health restrictions and childcare practices varied considerably across time and in different settings. Children in the pandemic cohort experienced their early childhood during the COVID-19 pandemic and had been exposed to approximately one year of infection control restrictions before the baseline assessment was conducted.

Developmental outcomes were assessed at two time points, and hierarchical regression analyses were conducted to compare the two cohorts and examine whether the association between fine motor skills and vocabulary differed across these contrasting developmental contexts.

### 2.2. Motor and Language Development

Fine motor skills and language abilities were assessed using the Child Developmental Scale (CDS), which Anme et al. developed in 2007 [33]. The CDS structure provides age-specific developmental checkpoints at 2-month intervals for children aged 12–18 months, 3-month intervals between 18 and 48 months, and 6-month intervals thereafter. As part of the CDS assessment protocol, childcare professionals receive regular training and calibration, and evaluators must achieve at least 80% agreement with the established assessment standards before conducting child assessments. Previous validation studies reported reliability values of 92.9% and 82.5% for the fine motor and vocabulary domains, respectively [33]. Children were excluded from this study if they had diagnosed mental or physical disabilities or were missing data on the primary study variables. We also excluded children whose developmental age was three or more CDS levels below their chronological age at baseline, in accordance with the CDS assessment guidelines [33,34], along with those missing baseline data on fine motor skills or vocabulary.

### 2.3. Covariates

Drawing on previous research demonstrating the influence of demographic and family environmental factors on child development [20,21,22,35,36], the following variables were included as covariates in the regression models: age group (12–35 months = 0; 36–59 months = 1), gender (boy = 0; girl = 1), family structure (nuclear family = 0; extended family = 1), parental status (two-parent = 0; single-parent = 1), sibling presence (no = 0; yes = 1), and cohort (pre-pandemic = 0; pandemic = 1). Participants were categorized into two age groups (12–35 months and 36–59 months) based on the age-specific developmental assessment criteria used to evaluate developmental outcomes; therefore, age group was selected as the primary adjustment variable for the main analysis.

The home-rearing environment (HRE) was assessed using the Index of Child Care Environment (ICCE), a validated parental questionnaire developed by Anme et al. (2013) [35], demonstrating good criterion validity, with the total score showing a strong correlation with the Home Observation for Measurement of the Environment (HOME) inventory (*r* = 0.76) and high inter-rater reliability (reproducibility coefficient = 0.91) [35]. Following the original scoring procedure, higher scores indicated a more supportive home-rearing environment [20,21,35,36], and the total ICCE score was included in the models as a continuous variable.

### 2.4. Statistical Analysis

Descriptive statistics were calculated for all study variables. Mann–Whitney U tests were used to compare study variables between cohorts.

Hierarchical regression analyses were conducted to examine the predictors of vocabulary at T2. In Step 1, baseline vocabulary, gender, and age group were entered; in Step 2, family structure, parental status, sibling presence, and HRE (ICCE total score); and in Step 3, baseline fine motor skills, cohort, and the interaction term between baseline fine motor skills and cohort to examine whether the motor skill and vocabulary association differed across cohorts. For the sensitivity analysis, the categorical age group variable was replaced with chronological age (months) as a continuous covariate, and the results of this analysis are presented in Supplementary Table S1.

Prior to conducting the hierarchical regression analyses, standard statistical assumptions were verified. The normality of the residuals, linearity, and homoscedasticity were confirmed through visually inspecting residual plots, and multicollinearity was checked using the Variance Inflation Factor (VIF), with all values falling well below the acceptable threshold of 10. For all analyses, including the two-way interaction, the significance threshold was set at alpha = 0.05. Additionally, a post hoc power analysis conducted using G*Power (Version 3.1) indicated that with our sample size (*n* = 1150) and an alpha level of 0.05, the statistical power to detect the interaction effect was 1.00, confirming that this study had sufficient power.

When a significant interaction was identified, the Johnson–Neyman technique was applied to determine the regions of significance across the fine motor skill score range [37].

Missing values occurred primarily in the vocabulary scores at T2, with the overall proportion of missing data across all analyzed variables remaining less than 1%. These values were handled using multiple imputation (fully conditional specification) with 30 imputed datasets under the assumption of missing at random (MAR). Primary regression analyses were performed using IBM SPSS Statistics (Version 30), and the PROCESS macro (Version 5.0) and Johnson–Neyman analyses based on the multiply imputed datasets were conducted manually in Mplus (Version 8.3). Statistical significance was set at *p* < 0.05 [38,39].

## 3. Results

**Figure 1:**
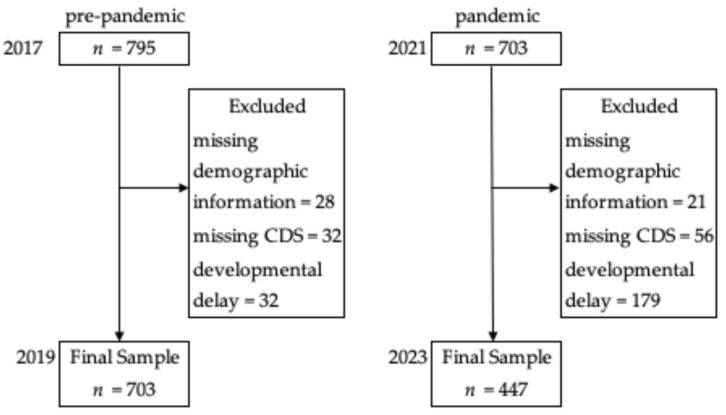
Participant flow chart.

The participant selection process is shown in Figure 1. The final analytical sample consisted of 1150 children, including 703 in the pre-pandemic and 447 in the pandemic cohort. The demographic characteristics are presented in Table 1.

The baseline characteristics are presented in Table 2. Compared with the pre-pandemic cohort, children in the pandemic cohort were significantly older at baseline and demonstrated higher baseline vocabulary and fine motor scores (all *p* < 0.01). In contrast, no significant difference was observed in their home-rearing environment, as measured by the total ICCE score (*p* = 0.923).

### 3.1. Hierarchical Regression Analysis

A three-step hierarchical regression analysis was conducted to predict vocabulary at T2 (Table 3).

The baseline vocabulary, gender, and age group were entered into Model 1, which explained 64.5% of the variance in T2 vocabulary (*R*^2^ = 0.645, *p* < 0.001). Baseline vocabulary (*B* = 0.80, *SE* = 0.04, 95% CI [0.72, 0.87], *p* < 0.001), gender (*B* = 2.32, *SE* = 0.59, 95% CI [1.17, 3.48], *p* < 0.001), and age group (*B* = 6.11, *SE* = 1.01, 95% CI [4.14, 8.10], *p* < 0.001) were significant predictors.

Parental status, family structure, sibling presence, and home-rearing environment (ICCE) were included in Model 2, though they did not significantly improve the model fit (Δ*R*^2^ = 0.003, *p* = 0.056), resulting in a cumulative *R*^2^ of 0.648.

T1 fine motor skills, cohort, and the interaction between the two were included in Model 3. This step explained an additional 2.5% of the variance (Δ*R*^2^ = 0.025, *p* < 0.001), yielding a total *R*^2^ of 0.673. Fine motor skills (*B* = 0.50, *SE* = 0.06, 95% CI [0.39, 0.61], *p* < 0.001) and cohort (*B* = −1.83, *SE* = 0.59, 95% CI [−2.98, −0.67], *p* = 0.002) were significant predictors, and a significant interaction between fine motor skills and cohort was observed (*B* = −0.09, *SE* = 0.05, 95% CI [−0.18, −0.00], *p* = 0.041). The sensitivity analysis yielded findings consistent with the primary analyses, indicating that the main interaction effects remained significant when age was modeled as a continuous covariate (Supplementary Table S1).

### 3.2. Moderation Analysis

**Figure 2:**
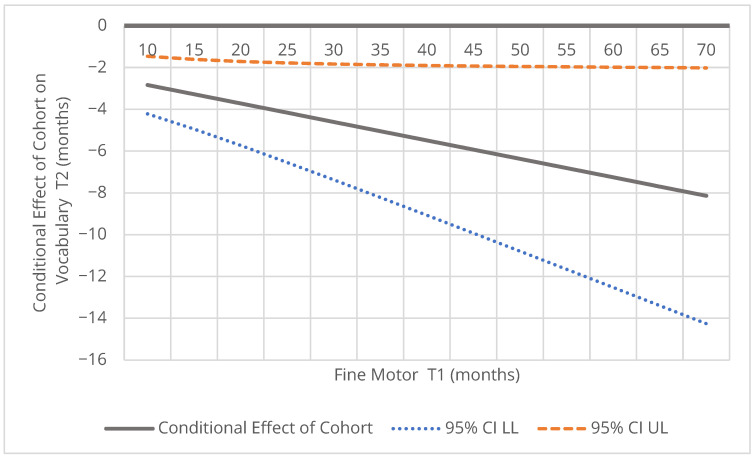
Conditional effect of being in the COVID-19 cohort on vocabulary as a function of fine motor skills. Note: T1 (pre-pandemic: 2017; pandemic: 2021) and T2 (pre-pandemic: 2019; pandemic: 2023). CI: Confidence Interval. LL: Lower Limit. UL: Upper Limit.

Following the significant interaction identified in the hierarchical regression analysis (Table 3), a Johnson–Neyman analysis was conducted to further examine the conditional effect of cohort (Figure 2), which was significant across the entire observed range of T1 fine motor skills. At all baseline fine motor skill levels, children in the pandemic cohort had significantly lower T2 vocabulary scores than those in the pre-pandemic cohort with comparable T1 fine motor skills. Moreover, the difference between the cohorts increased as baseline fine motor skills increased.

## 4. Discussion

In the present study, we longitudinally examined whether the association between early fine motor skills and later vocabulary growth differed before and during the COVID-19 pandemic. Consistent with previous research, baseline fine motor skills were positively associated with later vocabulary development in both cohorts [15,16,17,18,22], suggesting that the motor–language association remained evident despite the substantial social and environmental changes associated with the pandemic.

Simultaneously, the strength of this association differed between cohorts. After adjusting for age group, children in the pandemic cohort demonstrated significantly lower vocabulary outcomes than those in the pre-pandemic cohort across the entire observed range of fine motor proficiencies. Furthermore, this difference became more pronounced as baseline fine motor skills increased. Specifically, the Johnson–Neyman analysis revealed that while children with lower baseline fine motor skills (e.g., at the 25-month skill level) experienced a cohort-related developmental deficit of approximately four months in later vocabulary level, those with advanced baseline skills (e.g., at the 70-month skill level) demonstrated a larger deficit of approximately eight months. These findings remained robust after adjusting for age group, suggesting that the observed cohort differences were not attributable solely to differences in age composition. To better understand this pattern, it is important to consider the potential mechanisms underlying the weaker motor–language association observed in the pandemic cohort.

One hypothetical explanation is that changes in children’s communicative environments during the COVID-19 pandemic contributed to the weaker association observed in the pandemic cohort. Government guidance during this time recommended infection control measures such as the use of masks by childcare professionals, acrylic clear panels, social distancing, and mokushoku [9,40]. Consistent with these environmental changes, studies conducted in French and Japanese childcare centers have reported that educators perceived reductions in verbal communication among children under three years of age when interacting with masked adults, including less vocalization, repetition, and spontaneous verbal expression [41]. Previous research has shown that language development is supported through socially embedded experiences, including imitation, joint attention [42], caregiver–child interactions [43,44], and spontaneous verbal exchanges [45,46,47,48]. In addition, opportunities for group expressive activities were reduced or modified during the pandemic [9]. Together, these environmental changes may have been associated with fewer opportunities for children to integrate motor experiences into their language learning [40,45]. From the perspective of Bronfenbrenner’s ecological theory [5,6], these findings are consistent with the possibility that pandemic-related changes in children’s daily environments are associated with differences in early learning experiences. However, because the present study did not directly assess children’s exposure to these environmental conditions, these interpretations should be regarded as hypotheses requiring further research.

Furthermore, the findings should be interpreted within the unique context of Japan, where licensed childcare facilities largely remained open with rigorous infection control measures throughout the COVID-19 pandemic [9]. Continuous access to childcare may have provided opportunities for social interaction and structured learning that helped to preserve children’s developmental trajectories [30], a context which may partly explain why the longitudinal association between fine motor skills and vocabulary remained evident despite pandemic-related disruptions.

In contrast, a clinical study from Turkey comparing children before and during the COVID-19 pandemic reported increased language delays but reduced fine motor delays when developmental domains were examined independently using the Denver II Developmental Screening Test [49]; however, unlike this research, that study did not investigate the longitudinal association between early fine motor skills and later vocabulary development. Differences in study design and participant characteristics, including clinical sample use, may have contributed to these differing findings. Future studies should investigate whether motor–language associations differ among children with neurodevelopmental conditions and other clinical populations.

The present findings have important implications for early childhood education and childcare practices. Sensitivity analyses (Supplementary Table S1), in which chronological age was modeled as a continuous covariate, yielded findings consistent with the primary analyses. Although the main effect of fine motor skills was reduced after continuous adjustment for age, the interaction between the cohort and fine motor skills remained significant, supporting the robustness of observed cohort differences. These findings suggest that the longitudinal motor–language association should not be considered an isolated or automatic process; the benefits of fine motor experiences may depend on the broader social and communicative environments in which they occur. Therefore, early childhood educators and caregivers should intentionally integrate fine motor activities with responsive communication, shared play, and meaningful verbal exchanges to support children’s language development. Activities such as drawing, block play, arts and crafts, and object manipulation provide valuable opportunities to foster motor and language development in daily practice.

Overall, the present findings suggest that the environmental context may influence longitudinal motor–language association strength while preserving the fundamental developmental relationships between these domains. Although the interaction effect accounted for a relatively modest proportion of the variance in vocabulary development (Δ*R*^2^ = 0.025), even small differences in developmental associations may be meaningful at the population level because they reflect changes affecting children across a broad range of developmental abilities. To our knowledge, previous studies primarily examined changes within individual developmental domains, whereas the present study is among the first to investigate whether the longitudinal association between early fine motor skills and later vocabulary development differed before and during the COVID-19 pandemic.

## 5. Limitations and Future Directions

Several limitations should be considered when interpreting the findings of this study. First, although the longitudinal design provides insight into temporal sequences, the observational nature precludes causal inferences. Although fine motor skills predict later vocabulary, the mechanisms underlying this association have not been directly examined.

Second, the sample was drawn exclusively from licensed childcare centers in Japan; therefore, the findings may not be generalizable to countries that experienced prolonged childcare closures or strict lockdowns, where children’s developmental experiences differed substantially. This limitation should be considered when interpreting the findings in terms of the different educational and policy contexts.

Third, developmental outcomes and home-rearing environments were assessed through reports and may have therefore been subject to reporting bias. Although childcare professionals completed systematic training and achieved acceptable inter-rater agreement before conducting assessments, reporting bias cannot be ruled out in this study. Additionally, participation was limited to families enrolled in licensed childcare facilities, which may have introduced selection bias and limited the generalizability of our findings.

Fourth, small but statistically significant age differences were observed between the cohorts at both assessment points; however, sensitivity analyses using chronological age in months instead of categorical age groups yielded comparable cohort and interaction effect patterns, indicating that the interpretation of the main findings was unchanged (Supplementary Table S1).

Despite these limitations, in this study we provide longitudinal evidence of the association between fine motor skills and vocabulary development across two cohorts that experienced early development under different socio-environmental conditions. Future research should investigate the mechanisms underlying motor–language associations by incorporating direct measures of children’s everyday social and communicative environments. Replication in diverse cultural contexts and child populations, including neurodivergent children, would further enhance our understanding of how environmental conditions influence children’s early developmental processes.

## 6. Conclusions

In this study, we provide longitudinal evidence that fine motor skills are positively associated with later expressive vocabulary, both before and during the COVID-19 pandemic. Although the fundamental developmental relationship remained evident across cohorts, it was weaker in the pandemic cohort, suggesting that changes in children’s social and communicative environments during the pandemic may have influenced its strength. By demonstrating that the environmental context may modify how motor experiences contribute to language development, we extend the current understanding of early childhood developmental cascades. These findings underscore the importance of providing rich communicative experiences and opportunities for fine motor development in early childhood education and childcare settings, particularly during periods of social and environmental disruption.

## Figures and Tables

**Table 1:** Demographic information.

		Full Sample		Pre-Pandemic		Pandemic	
		*n*	%	*n*	%	*n*	%
Gender	Boy	587	51.0	369	52.5	218	48.8
	Girl	563	49.0	334	47.5	229	51.2
Parental Status	Two-parent	990	86.1	612	87.1	378	84.6
	Single-parent	160	13.9	91	12.9	69	15.4
Family Structure	Nuclear	1017	88.4	616	87.6	401	89.7
	Extended	133	11.6	87	12.4	46	10.3
Sibling Presence	No	485	42.2	264	37.6	221	49.4
	Yes	665	57.8	439	62.4	226	50.6

Note: *n* = 1150 (pre-pandemic *n* = 703; pandemic *n* = 447).

**Table 2:** Comparison of baseline T1 characteristics between the pre-pandemic and pandemic cohorts.

	Pre-Pandemic	Pandemic		
	*n* = 703	*n* = 447		
	*M ± SD*	*M* ± SD	z	*p*
Age (months)	32.71 ± 12.48	35.44 ± 11.66	4.03	<0.001
Vocabulary (months)	34.33 ± 13.69	37.00 ± 12.79	2.92	0.003
Fine Motor (months)	34.70 ± 13.22	37.16 ± 12.59	3.09	0.002
ICCE	11.72 ± 1.25	11.78 ± 1.11	0.10	0.923

Note: T1 (pre-pandemic: 2017; pandemic: 2021). ICCE: The Index of Child Care Environment.

**Table 3:** Hierarchical regression analysis predicting T2 vocabulary.

	Model 1		Model 2		Model 3	
	*B* (*SE*)	95% CI	*B* (*SE*)	95% CI	*B* (*SE*)	95% CI
(Constant)	22.97 (1.00) ***	[21.00, 24.94]	21.79 (3.09) ***	[15.73, 27.84]	18.50 (3.08) ***	[12.45, 24.54]
Vocabulary (T1)	0.80 (0.04) ***	[0.72, 0.87]	0.80 (0.04) ***	[0.73, 0.87]	0.46 (0.06) ***	[0.35, 0.56]
Gender	2.32 (0.59) ***	[1.17, 3.48]	2.34 (0.59) ***	[1.19, 3.50]	2.44 (0.57) ***	[1.33, 3.56]
Age Group	6.11 (1.01) ***	[4.14, 8.10]	5.98 (1.01) ***	[4.01, 7.96]	3.87 (1.00) ***	[1.90, 5.83]
Parental Status			−0.82 (0.88)	[−2.48, 0.94]	−0.54 (0.85)	[−2.19, 1.12]
Family Structure			−0.39 (0.91)	[−2.18, 1.40]	−0.57 (0.88)	[−2.30, 1.16]
Sibling Presence			1.51 (0.60) *	[0.33, 2.68]	1.02 (0.58)	[−0.12, 2.17]
ICCE			0.05 (0.25)	[−0.44, 0.53]	0.21 (0.24)	[−0.26, 0.68]
Fine Motor (T1)					0.50 (0.06) ***	[0.39, 0.61]
Cohort					−1.83 (0.59) **	[−2.98, −0.67]
Fine Motor (T1) × Cohort					−0.09 (0.05) *	[−0.18, −0.00] ^†^
*R* ^2^	0.645 ***		0.648		0.673 ***	
Δ*R*^2^	0.645		0.003		0.025	

Note: *n* = 1150. *B*: Unstandardized regression coefficients. *SE*: Standard Error. CI: Confidence Interval. T1 (pre-pandemic: 2017; pandemic: 2021) and T2 (pre-pandemic: 2019; pandemic: 2023). ICCE: The Index of Child Care Environment. * *p* < 0.05, ** *p* < 0.01, *** *p* < 0.001. ^†^ Negative values rounded to zero after rounding to two decimal places.

## Data Availability

The data presented in this study are not publicly available because of privacy and ethical restrictions.

## Supplementary Materials

The following supporting information can be downloaded at: https://www.mdpi.com/article/10.3390/children13070920/s1, Table S1. Sensitivity Analysis for Hierarchical Regression Predicting T2 Vocabulary: Age as a Continuous Variable.
