# Supplementary material for: Fine Motor Skills and Later Vocabulary Development Before and During the COVID-19 Pandemic: Evidence from Japanese Childcare Settings
Source: Children (Basel). 2026 Jul 12;13(7):920. doi: 10.3390/children13070920 (PMC13406418; doi:10.3390/children13070920)
Supplement: Supplementary file 1 [file children-13-00920-s001.zip › children-4395410-supplementary.pdf]

Table S1. Sensitivity Analysis for Hierarchical Regression Predicting T2 Vocabulary: Age as a Continuous Variable

	Model 1		Model 2		Model 3	
	B (SE)	95% CI	*B (SE)*	95% CI	*B (SE)*	95% CI
(Constant)	21.85 (1.01)***	[20.21, 23.50]	19.23 (2.84)***	[13.67, 24.80]	18.21 (2.89)***	[12.53, 23.88]
Vocabulary (T1)	0.32 (0.05)***	[0.23, 0.41]	0.32 (0.05)***	[0.23, 0.42]	0.431 (0.05)***	[0.20, 0.41]
Gender	3.22 (0.55)***	[2.15, 4.29]	3.21 (0.55)***	[2.14, 4.28]	3.25 (0.54)***	[2.18, 4.31]
Age	0.80 (0.05)***	[0.70, 0.90]	0.80 (0.05)***	[0.70, 0.90]	0.78 (0.07)***	[0.65, 0.91]
Parental Status			-0.66 (0.81)	[-2.25, 0.93]	-0.59 (0.80)	[-2.17, 0.98]
Family Structure			-0.64 (0.85)	[-2.31, 1.03]	-0.73 (0.84)	[-2.39, 0.92]
Presence of Siblings			1.20 (0.56)*	[0.11, 2.29]	0.87 (0.55)	[-0.21, 1.96]
ICCE			0.18 (0.23)	[-0.27, 0.64]	0.24 (0.23)	[-0.21, 0.69]
Fine Motor (T1)					0.08 (0.07)	[-0.05, 0.20]
Cohort					-2.31 (0.56)***	[-3.41, -1.22]
Fine Motor(T1) x Cohort					-0.09 (0.04)*	[-0.17, -0.00†]
R^2	.698***		.700		.706***	
ΔR^2	.668		.002		.006	

Note: N = 1150 *B*: Unstandardized regression coefficients. *SE*: Standard Error. CI: Confidence Interval. T1 (pre-pandemic: 2017, pandemic: 2021) and T2 (pre-pandemic: 2019; pandemic: 2023). ICCE: the Index of Child Care Environment. **p*<.05, ***p*<.001, ****p*<.0001. † Negative values rounded to zero after rounding to two decimal places.
